# Presynaptic BK channel localization is dependent on the hierarchical organization of alpha-catulin and dystrobrevin and fine-tuned by CaV2 calcium channels

**DOI:** 10.1186/s12868-015-0166-2

**Published:** 2015-04-24

**Authors:** Kelly H Oh, Linu S Abraham, Chandler Gegg, Christian Silvestri, Yung-Chi Huang, Mark J Alkema, Jacob Furst, Daniela Raicu, Hongkyun Kim

**Affiliations:** Department of Cell Biology & Anatomy, Chicago Medical School, Rosalind Franklin University, 60064 North Chicago, IL USA; College of Computing and Digital Media, DePaul University, 60604 Chicago, IL USA; Department of Biology, Lake Forest College, 60045 Lake Forest, IL USA; Department of Neurobiology, University of Massachusetts Medical School, 01605 Worcester, MA USA

**Keywords:** *C. elegans*, SLO-1/BK potassium channels, UNC-2/CaV2 calcium channels, CTN-1/α-catulin, DYB-1/dystrobrevin

## Abstract

**Background:**

Large conductance, calcium-activated BK channels regulate many important physiological processes, including smooth muscle excitation, hormone release and synaptic transmission. The biological roles of these channels hinge on their unique ability to respond synergistically to both voltage and cytosolic calcium elevations. Because calcium influx is meticulously regulated both spatially and temporally, the localization of BK channels near calcium channels is critical for their proper function. However, the mechanism underlying BK channel localization near calcium channels is not fully understood.

**Results:**

We show here that in *C. elegans* the localization of SLO-1/BK channels to presynaptic terminals, where UNC-2/CaV2 calcium channels regulate neurotransmitter release, is controlled by the hierarchical organization of CTN-1/α-catulin and DYB-1/dystrobrevin, two proteins that interact with cortical cytoskeletal proteins. CTN-1 organizes a macromolecular SLO-1 channel complex at presynaptic terminals by direct physical interaction. DYB-1 contributes to the maintenance or stabilization of the complex at presynaptic terminals by interacting with CTN-1. We also show that SLO-1 channels are functionally coupled with UNC-2 calcium channels, and that normal localization of SLO-1 to presynaptic terminals requires UNC-2. In the absence of UNC-2, SLO-1 clusters lose the localization specificity, thus accumulating inside and outside of presynaptic terminals. Moreover, CTN-1 is also similarly localized in *unc-2* mutants, consistent with the direct interaction between CTN-1 and SLO-1. However, localization of UNC-2 at the presynaptic terminals is not dependent on either CTN-1 or SLO-1. Taken together, our data strongly suggest that the absence of UNC-2 indirectly influences SLO-1 localization via the reorganization of cytoskeletal proteins.

**Conclusion:**

CTN-1 and DYB-1, which interact with cortical cytoskeletal proteins, are required for the presynaptic punctate localization of SLO-1 in a hierarchical manner. In addition, UNC-2 calcium channels indirectly control the fidelity of SLO-1 puncta localization at presynaptic terminals. We suggest that the absence of UNC-2 leads to the reorganization of the cytoskeletal structure that includes CTN-1, which in turn influences SLO-1 puncta localization.

**Electronic supplementary material:**

The online version of this article (doi:10.1186/s12868-015-0166-2) contains supplementary material, which is available to authorized users.

## Background

Calcium influx through voltage-gated calcium channels (VGCCs) regulates essential physiological functions in excitable cells, including muscle excitation-contraction, hormone release, synaptic transmission and gene expression [1]. However, unregulated excessive calcium influx is harmful to the cells and is postulated to be a cause of many degenerative diseases. To protect against inadvertent calcium overload, VGCCs have built-in failsafe regulatory mechanisms, including channel inactivation through voltage- and calcium-dependent conformational changes [2]. Another physiologically important negative regulator of VGCCs in excitable cells is large conductance, calcium- and voltage-dependent potassium (BK) channels [3]. In addition to a voltage-sensitive ion channel domain, BK channels possess low-affinity (3 ~ 50 μM) calcium-binding sites in their C-terminal cytoplasmic domain [4]. Calcium binding at these sites induces a conformational change in the gate ring and increases the open probability of the pore [5,6]. Hence, rapid and effective activation of the BK channel requires not only membrane depolarization but also elevations in free cytosolic calcium ions. Once BK channels are activated, they feed back onto intracellular calcium increases by curbing calcium influx through the deactivation of VGCCs [3]. Because calcium influx into cells is temporally and spatially controlled, calcium concentrations reach the levels required for BK channel activation only near the vicinity of calcium channels. For this reason, BK channels are localized in calcium nanodomains where calcium channels are localized [7,8]. Despite the critical link between the localization of BK channels and their function as a calcium channel regulator, the mechanism by which BK channels localize to calcium nanodomains is not well understood.

In *C. elegans,* SLO-1/BK channels are found near dense bodies in muscles, near where L-type EGL-19/CaV1 calcium channels are localized [9], and at presynaptic terminals, where UNC-2/CaV2 calcium channels are localized [10]. *C. elegans* genetic studies by our groups, as well as by others, demonstrated that SLO-1 localization is controlled by components of the dystrophin complex that interacts with cortical cytoskeletal proteins and is associated with several different forms of muscular dystrophy [11]. Interestingly, SLO-1 localization in muscle and neurons requires shared but slightly different mechanisms. Notably, although two conserved cytoskeleton-interacting proteins, DYB-1/dystrobrevin and CTN-1/α-catulin, are necessary for SLO-1 localization in both muscle and neurons, DYS-1/dystrophin, which physically interacts with DYB-1 and CTN-1, is essential for SLO-1 localization in muscle but not in neurons [12].

In this study, we show that SLO-1 localization at presynaptic terminals is controlled by the hierarchical organization of CTN-1 and DYB-1; SLO-1 channels are localized by CTN-1, which is recruited to presynaptic terminals by DYB-1. Furthermore, we show that the presynaptic localization of SLO-1 channels does not require UNC-2, but the strict localization of SLO-1 channels to presynaptic terminals does require the presence of UNC-2. In the absence of UNC-2, CTN-1 and SLO-1 localizations become dispersed throughout presynaptic regions.

## Results

### CTN-1/α-catulin interacts with SLO-1/BK via two RCK (regulator of potassium conductance) domains

**Figure 1:**
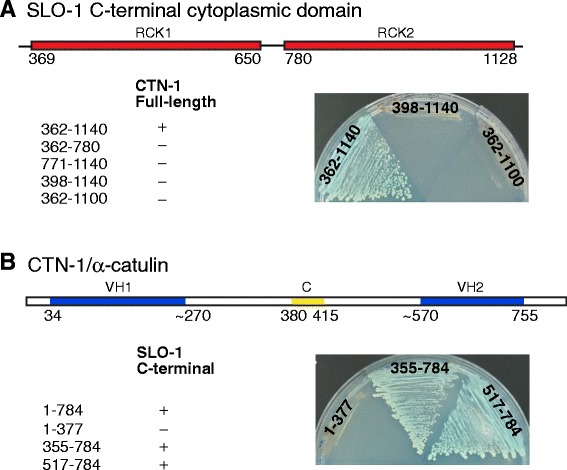
Yeast two-hybrid assays reveal the interaction between CTN-1/α-catulin and the two RCK domains of SLO-1/BK. **(A)** Both the RCK1 and RCK2 domains of SLO-1 are necessary for the interaction with CTN-1. The indicated portions of the SLO-1 C-terminal region were fused in frame to the GAL4 activation domain and tested for interaction with CTN-1 tagged with the GAL4 DNA-binding domain. Images obtained from different plates cultured under identical conditions are stitched together for the simplicity of presentation. **(B)** The C-terminal region of CTN-1 that encompasses the VH2 domain interacts with SLO-1. The indicated portions of CTN-1 were fused in frame to the GAL4-DNA binding domain and tested for interaction with the SLO-1 C-terminal region tagged with the GAL4 activation domain. VH1, vinculin homology domain 1; C, coiled-coil domain; VH2, vinculin homology domain 2.

Previous studies by us and others showed that mutations in *ctn-1*, which displays extensive homology to both α-catenin and vinculin, suppress the sluggish movement of *slo-1* gain-of-function mutants [12], and that SLO-1 interacts with CTN-1 in *C. elegans* muscle and in heterologous cultured cells [13]. However, the physical and functional interaction between SLO-1 and CTN-1 in neurons has not been fully examined. As a first step, we sought to determine the exact domains of SLO-1 and CTN-1 that are necessary for their physical interaction by a yeast two-hybrid assay. The cytoplasmic tail of SLO-1 consists of two RCK domains (regulator of K^+^ conductance), which bind calcium ions, and a linker sequence, which is variable among different isoforms but is not essential for structural integrity [4]. Intriguingly, no interaction with CTN-1 was observed with either RCK domain alone, but instead required both RCK domains simultaneously (Figure 1A). Deleting the N-terminal 36 amino acids of the RCK1 domain or the C-terminal 40 amino acids of the RCK2 domain disrupted the interaction with CTN-1. Similarly mapping the SLO-1 interaction region of CTN-1 revealed that the C-terminal region of CTN-1 was required for SLO-1 channel interaction (Figure 1B). Because we previously showed that the N-terminal region of CTN-1 interacts with DYB-1 [14], these results indicate that CTN-1 can interact with SLO-1 and DYB-1.

### SLO-1/BK localizes to the presynaptic terminals via the hierarchical organization of CTN-1/α-catulin and DYB-1/dystrobrevin

**Figure 2:**
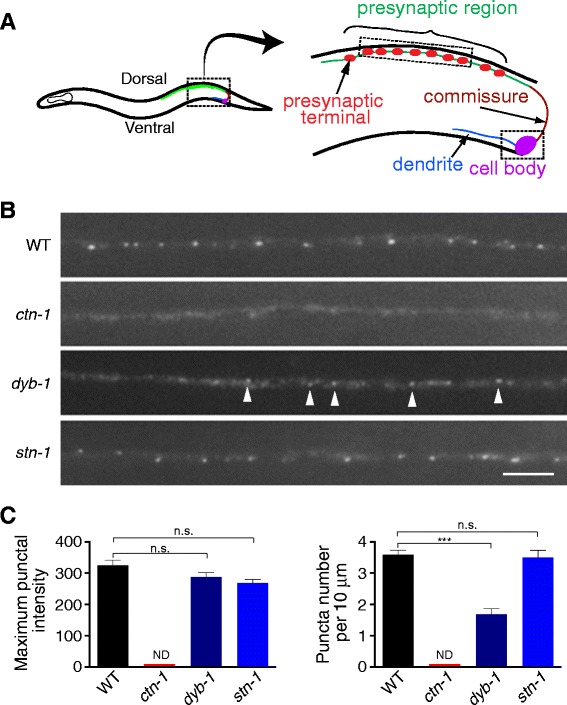
SLO-1/BK localization at presynaptic terminals is controlled by CTN-1/α-catulin and DYB-1/dystrobrevin, but not by STN-1/syntrophin. **(A)** Schematic representation of the anatomical structure of a DA neuron. The DB neurons have an axon and dendrites that extend in the other direction. The *unc-129* promoter (~2. 4 kb) drives expression in a subset of DA and DB cholinergic motor neurons. The axon processes with neuromuscular and VD synapses (*green*) are presynaptic regions, and individual synaptic areas (*red*) are presynaptic terminals. **(B)** Representative images of SLO-1::GFP in the axon terminals of DA and DB neurons in wild-type, *ctn-1*, *dyb-1* and *stn-1* mutant animals. Arrowheads highlight SLO-1::GFP puncta. The scale bar represents 5 μm. **(C)** Quantification of SLO-1::GFP puncta in wild-type and mutant animals. The maximum intensity of puncta and the number of puncta within a 10 μm distance were calculated using dotGUI (see Methods). For all the panels, data are presented as the mean ± SEM. *** and n.s. indicate a statically significant difference (multiplicity adjusted *p* < 0.001) and no significant difference between indicated groups, respectively (One-way ANOVA Dunett’s multiple comparison. wild-type, n = 11; *dyb-1,* n = 12; *stn-1;* n = 5). ND, not determined.

To precisely visualize SLO-1 localization at the presynaptic region, we used transgenic animals expressing GFP-tagged SLO-1 under the *unc*-*129* promoter (Figure 2A). The *unc-129* promoter (2.4 kb from the translational initiation codon) drives expression in DA and DB cholinergic motor neurons (9 neurons) whose cell bodies are located at the ventral cord [15,16]. The dendrites of these neurons are localized along the ventral cord, and the axons travel circumferentially along the hypodermis to reach the dorsal cord, where they project longitudinally to form *en passant* synapses with striated body muscle and VD motor neurons [17]. Such spatially separated axon commissures, synaptic terminals and dendrites allow easy distinction among different neuronal compartments. In wild-type animals, SLO-1::GFP exhibited bright, tiny punctate structures with weak diffuse fluorescence along the presynaptic regions of DA and DB neurons, but not in the dendrite regions (Figure 2, see below). SLO-1::GFP puncta colocalized with the synaptic vesicle marker RAB-3::mCherry (see below). Therefore, it is most likely that SLO-1::GFP puncta represent presynaptic terminals (Figure 2A).

Next, we examined if SLO-1 localization to presynaptic terminals is affected by mutations in *ctn-1* and *dyb-1*. In *ctn-1* mutants, SLO-1 did not show any punctate pattern but was broadly expressed in the presynaptic regions of both DA and DB neurons (Figure 2B, C). In fact, because *ctn-1* mutants did not show discrete SLO-1::GFP puncta, we were not able to calculate maximum punctal intensity and inter-punctal distance in *ctn-1* mutants. These results indicate that *ctn-1* is required for localizing or organizing SLO-1 at presynaptic terminals, but not for transporting SLO-1 to the presynaptic regions. Supporting the role of CTN-1 in organizing SLO-1 at presynaptic terminals, we also found that when SLO-1::GFP and mCherry::CTN-1 are co-expressed in DA and DB motor neurons, they are co-localized (Additional file 1: Figure S1). In *dyb-1* mutants, SLO-1::GFP puncta density at the presynaptic region was greatly reduced, but not as severely as in *ctn-1* mutants (Figure 2B,C). However, the intensity of individual SLO-1::GFP puncta appeared not to be significantly different from that of wild-type control animals (Figure 2B,C). Several studies in mammals and *C. elegans* demonstrated that DYB-1/dystrobrevin can physically interact with STN-1/syntrophin [18,19]*.* Thus, we examined whether SLO-1 synaptic localization is altered in *stn-1* mutants. We found that SLO-1 localization at the synaptic terminals remained intact in *stn-1* mutants (Figure 2B,C), indicating that STN-1 does not critically contribute to the localization of SLO-1 to presynaptic terminals. Importantly, altered SLO-1 punctal patterns in *ctn-1* and *dyb-1* mutants do not appear to result from a decrease in SLO-1 expression in the presynaptic regions. When we quantified the overall SLO-1 fluorescence intensity along the presynaptic region, we found that wild-type, *ctn-1* and *dyb-1* animals do not exhibit any obvious difference (Additional file 2: Figure S2).

**Figure 3:**
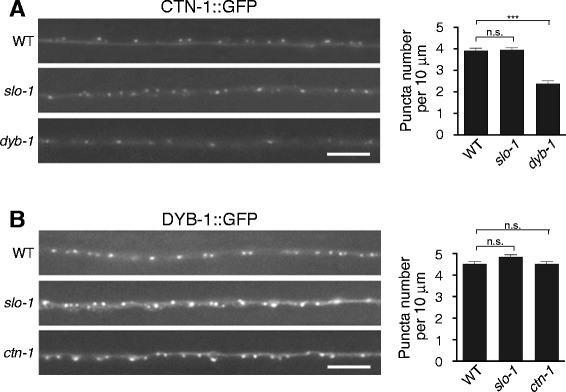
Presynaptic localization of CTN-1/α-catulin and DYB-1/dystrobrevin **(A)** Presynaptic localization of CTN-1/α-catulin is partially dependent on DYB-1/dystrobrevin. The integrated array *cimIs8,* which drives GFP-tagged CTN (GFP::CTN-1) expression in a subset of DA and DB neurons, was crossed to *slo-1* or *dyb-1* mutants. The number of puncta within 10 μm in each data point was presented as the mean ± SEM and analyzed by one-way ANOVA Dunett’s multiple comparison (wild-type, n = 20; *slo-1*, n = 29; *dyb-1,* n = 20. ***p < 0.001; n.s. *p* > 0.05). **(B)** Presynaptic localization of DYB-1/dystrobrevin is independent of CTN-1/α-catulin. The integrated array *cimIs15,* which drives GFP-tagged DYB (GFP::DYB-1) expression in a subset of DA and DB neurons, was crossed to *slo-1* or *ctn-1* mutants. The number of puncta within 10 μm in each data point was presented as the mean ± SEM (one-way ANOVA Dunett’s multiple comparison, wild-type, n = 34; *slo-1,* n = 31; *ctn-1*, n = 30; n.s. *p* > 0.05). The scale bar represents 5 μm.

To further explore the interplay between SLO-1, CTN-1 and DYB-1 at the presynaptic regions, we examined the presynaptic localization of CTN-1 and DYB-1 in DA and DB neurons. Consistent with the role of CTN-1 in SLO-1 localization, CTN-1::GFP prominently localizes in a punctate pattern at presynaptic terminals (Figure 3A). The density of CTN-1 presynaptic puncta was significantly reduced in *dyb-1* mutants, but not in *slo-1* mutants. These results demonstrate that CTN-1 synaptic localization is at least partially dependent on DYB-1 but independent of SLO-1. Similarly to CTN-1, DYB-1::GFP prominently localizes to the synaptic terminals of DA and DB neurons (Figure 3B). We found that DYB-1 presynaptic localization was not significantly affected in either *slo-1* or *ctn-1* mutants. These results indicate that SLO-1, CTN-1 and DYB-1 are hierarchically organized; SLO-1 presynaptic localization requires CTN-1, and CTN-1 presynaptic localization is partially dependent on DYB-1.

### An *unc-2*/CaV2α_1_ gain-of-function mutation suppresses the sluggish movement of *slo-1* gain-of-function mutants

**Figure 4:**
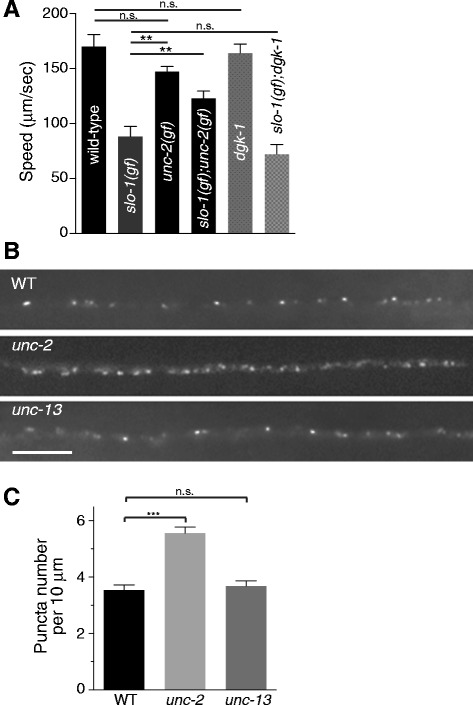
Genetic interaction of SLO-1/BK and UNC-2/CaV2 channels. **(A)** SLO-1/BK functionally interacts with UNC-2/CaV2 channels for locomotory behavior. A gain-of-function *unc-2(zf35)* mutation suppresses the sluggish movement of *slo-1(gf)* mutants, whereas *dgk-1* mutation, which causes a hyperactive phenotype, does not. The data are presented as the mean ± SEM and analyzed by one-way ANOVA with Bonferroni’s post Hoc test (**p < 0.01, n.s., not significant). **(B and C)** A loss-of-function *unc-2* mutation increases the SLO-1 punctal density in the presynaptic region. The number of SLO-1 puncta in a given length of the axonal terminal (i.e., decreased punctal distance) is higher in *unc-2* mutants than wild-type or *unc-13* animals. The quantification of SLO-1 puncta number was performed in *cimIs10* animals whose genetic background is wild-type, *unc-2* or *unc-13* animals. The data are presented as the mean ± SEM and analyzed by two-tailed Student t-test (wild-type, n = 14; *unc-2*, n = 11; *unc-13*, n = 12 ***p < 0.001, n.s., not significant). Scale bar, 5 μm.

BK channels and VGCCs co-purify from rodent brain extracts [20] and co-localize in Purkinje cells [21]. Furthermore, the physiological properties of BK channels can be modulated by their association with VGCCs [22]. Together these results indicate that BK channels and VGCCs are closely linked, both physically and functionally. Previous *C. elegans* genetic studies showed that *unc-2* (P/Q-type VGCC) and *slo-1* function together in a certain context at the same genetic pathway [23]. A genetic screen for altered asymmetric odorant receptor expression yielded *unc-2* and *slo-1(gf)* alleles. Further epistatic analysis showed that these two genes function upstream of *unc-43*, which encodes calcium/calmodulin dependent protein kinase II, but downstream of axon guidance mutants [24]. Given the noted functional coupling between BK channels and VGCCs in mammals and the genetic interaction in *C. elegans* gene expression, we investigated whether UNC-2 and SLO-1 are functionally coupled for *C. elegans* locomotion. Because *C. elegans* moves by generating and propagating sinuous undulations along the body axis, coordinated locomotion requires the precise temporal control of activation/inactivation cycles in presynaptic motor neurons. To investigate this functional coupling, we used a gain-of-function *slo-1(ky399gf)* mutant [23]. This mutant exhibits a sluggish locomotory behavior, which is attributed to delayed closing kinetics of the SLO-1 channel [25]. We reasoned that overly active SLO-1(gf) channels render VGCCs inactive via hyperpolarization, resulting in reduced excitability and synaptic release. In this case, we expected that ramping up VGCC function might overcome SLO-1(gf) inhibition. Therefore, we tested whether a gain-of-function mutation in the pore-forming α_1_ subunit, *unc-2(gf)* [26], can suppress the sluggish movement of *slo-1(gf)* mutants (Figure 4A and Additional file 3: MP4). We found that the *slo-1(ky399gf);unc-2(gf)* double mutant exhibits significantly improved movement compared to single *slo-1(gf)* mutants alone (Figure 4A). Given that *slo-1(gf)* mutants exhibit a reduced neurotransmitter release [12], one possibility is that general increase of synaptic function may nonspecifically suppress the sluggish movement of *slo-1(gf)* mutants. To test this possibility, we examined whether hyperactive mutant with increased synaptic function can suppress the sluggish *slo-1(gf)* movement. *dgk-1* mutant has a defect in a diacylglycerol kinase and has a hyperactive locomotion phenotype [27,28]. We found that *dgk-1* mutant cannot suppress the sluggish movement of *slo-1(gf)* mutants, providing evidence for the specificity of the interaction between *slo-1* and *unc-2* genes (Figure 4A). These results are consistent with the idea that the main function of SLO-1 in presynaptic terminals is to negatively regulate calcium channel function.

### UNC-2/CaV2 is required for the normal localization and distribution of SLO-1/BK at the presynaptic region

**Figure 5:**
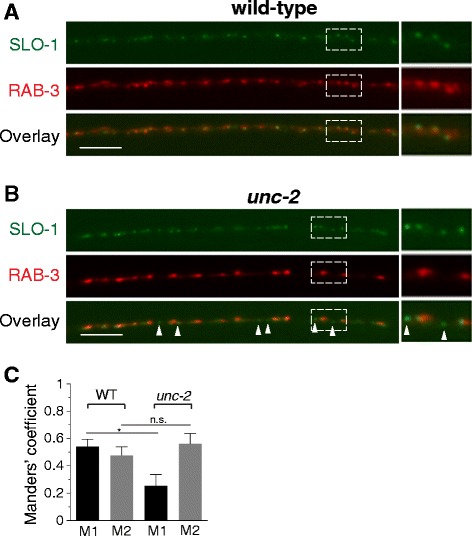
The organization of SLO-1/BK at the presynaptic terminals is defective in *unc-2* mutants. **(A, B)** An integrated SLO-1::GFP array *cimIs10* was crossed with wild-type **(A)** or *unc-2* **(B)** animals carrying the integrated RAB-3::mCherry array *cimIs14.* Arrowheads indicate SLO-1 puncta that are not co-localized with clusters of synaptic vesicles shown by RAB-3 puncta. The scale bars represent 5 μm. **(C)** Co-localization between SLO-1::GFP and RAB-3::mCherry in wild-type and *unc-2* animals was analyzed with JACoP, an ImageJ plugin [44]. Manders’ coefficient M1 is defined as the ratio of the summed intensity of GFP pixels overlapped with mCherry to total GFP intensity, whereas M2 is conversely defined. M1 coefficients in wild-type (n = 5) and *unc-2* (n = 4) animals are significantly different, whereas M2 coefficients in both animals are not different (Student t-test, n.s., *p* = 0.34; *p = 0.013). The data represent mean ± SEM.

In addition to their functional connection, a previous study suggested that BK channels and VGCCs are co-assembled together within calcium nanodomains [20]. Hence, it is possible that BK channel organization in presynaptic terminals is directly dependent on VGCCs [29]. VGCCs, particularly the N- and P/Q-types, are localized primarily at active zones [30-32]. If UNC-2 is critical for SLO-1 localization to presynaptic terminals, we expected that the localization of SLO-1 would be reduced or altered in *unc-2* mutants. Surprisingly, we found that the density of SLO-1 puncta was significantly increased in *unc-2* null mutants (Figure 4B, C). One potential explanation for this increased number of SLO-1 puncta at the presynaptic regions is that the overall expression level of SLO-1::GFP is increased in *unc-2* mutants. To test this possibility, we measured GFP levels in the cell bodies of DB6 and DA6 neurons. The levels of GFP in the cell bodies did not appear appreciably different between wild-type and *unc-2* mutant animals (Additional file 4: Figure S4). Thus, it is unlikely that additional ectopic SLO-1 puncta in *unc-2* mutant result from altered SLO-1::GFP expression levels. Another possibility is that reduction in presynaptic function in general, but not specifically in *unc-2* mutant, may disrupt SLO-1 localization at presynaptic terminals. To test this possibility, we examined SLO-1 localization in *unc-13* mutant, which has a defective synaptic vesicle fusion, thus greatly reduced synaptic transmission (more severely than *unc-2* mutant). We found that loss of *unc-13* function does not change the density of SLO-1 cluster in the presynaptic region (Figure 4B, C), indicating that general presynaptic defects do not disturb the presynaptic localization of SLO-1 clusters. To investigate whether these increased SLO-1 puncta correlate with synaptic vesicle release sites, we compared the co-localization pattern of SLO-1 and the synaptic vesicle marker RAB-3 in wild-type and *unc-2* mutant animals (Figure 5A). In wild-type animals, an average of one SLO-1 puncta was observed per synaptic vesicle cluster. In *unc-2* mutants, SLO-1 puncta were not only found within clusters of synaptic vesicles, but were also observed outside of synaptic vesicle clusters (Figure 5A, B, C), indicating that some SLO-1 puncta in *unc-2* mutants are present outside of neurotransmitter release sites (i.e., synaptic terminals). Importantly, the density of RAB-3::mCherry is not significantly different in wild-type and *unc-2* mutants (wild-type vs. *unc-2(lf)*, 4.0 ± 0.30 per 10 micron, n = 10 vs. 4.3 ± 0.27 per 10 micron, n = 9, *p* = 0.52, t-Test). As a way to address where extra SLO-1 puncta is originated in *unc-2* mutants, we compared SLO-1 punctal intensity in the presynaptic terminals of wild-type and *unc-2* animals, *unc-2* mutants exhibit a slight, but significant, reduction in average punctal intensity without change in overall SLO-1 levels in the presynaptic region (Additional file 5: Figure S5), suggesting that that extra SLO-1 puncta found in *unc-2* mutants may result from the dissociation or diffusion from unstable large puncta. Together, these results suggest that UNC-2 restricts SLO-1 clusters to presynaptic terminals.

**Figure 6:**
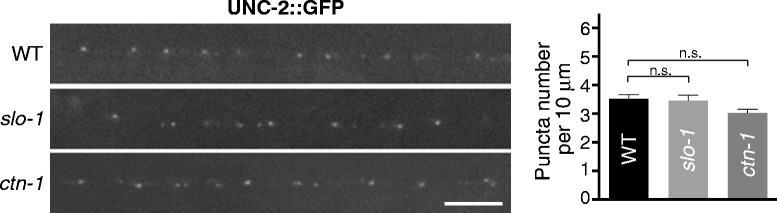
UNC-2/CaV2 localization is independent of SLO-1/BK localization. *slo-1* or *ctn-1* null mutants do not have any obvious defect in UNC-2 localization. Left panels: representative image. Right panels: quantification of the puncta number. Data were analyzed by one-way ANOVA with Dunnett’s multiple comparisons test (wt vs. *slo-1*: P = 0.94; wt vs. *ctn-1*: P = 0.07).

Because UNC-2 is required to restrict the localization of SLO-1 to presynaptic terminals, we examined whether SLO-1 or CTN-1 is conversely required for the normal UNC-2 localization to active zones. When we compared UNC-2 synaptic localization in wild-type and *slo-1* and *ctn-1* null mutants, we found that UNC-2 localization did not differ either between wild-type and *slo-1* animals or between wild-type and *ctn-1* animals (Figure 6). These results indicate that SLO-1 and CTN-1 do not have a role in the presynaptic localization of UNC-2.

**Figure 7:**
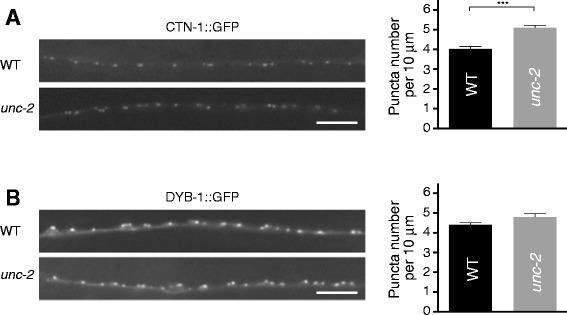
The disruption of SLO-1/BK localization in *unc-2* mutants is caused by altered CTN-1/α-catulin localization. **(A)** CTN-1 localization in wild-type and *unc-2* mutant animals. The number of GFP::CTN-1 puncta was increased in *unc-2* mutants compared with wild-type animals. The data are presented as the mean ± SEM and analyzed by two-tailed Student’s t-test (wild-type, n = 28; *unc-2*, n = 25; ***p < 0.001). **(B)** DYB-1 localization in wild-type and *unc-2* mutant animals. The number of GFP::DYB-1 puncta was slightly increased in *unc-2* mutants compared with wild-type animals. The data are presented as mean ± SEM and analyzed by two-tailed Student’s t-test (wild-type, n = 46; *unc-2*, n = 18; *p < 0.05). The scale bar represents 5 μm.

Based on our results showing that CTN-1 has a major role in organizing SLO-1 at presynaptic terminals (Figure 2A), we considered whether the abnormal pattern of SLO-1 puncta in *unc-2* mutants is caused by abnormal CTN-1 localization. When we compared CTN-1 puncta in wild-type and *unc-2* mutant animals, we indeed found that the number of CTN-1 puncta was similarly increased in *unc-2* mutants (Figure 7A). These results indicate that the defective SLO-1 localization in *unc-2* mutants is due to abnormal CTN-1 localization. Because CTN-1 localization is at least partially dependent on DYB-1, we determined whether DYB-1 localization is altered in *unc-2* mutants. We found that DYB-1 localization was minimally altered in *unc-2* mutants (Figure 7B). Together, these results indicate that CTN-1 localization is disrupted in *unc-2* mutants, and consequently, SLO-1 localization is compromised.

## Discussion

In the current study, we showed that SLO-1 localization in neurons is controlled by the hierarchical organization of CTN-1 and DYB-1. SLO-1 localizes to presynaptic terminals through direct interactions with CTN-1. The presynaptic localization of CTN-1 is partially dependent on DYB-1 because CTN-1 localization is disturbed by *dyb-1* mutation, but DYB-1 localization is not altered by *ctn-1* mutation. Surprisingly, we found that UNC-2/CaV2 is not required for SLO-1 localization at presynaptic terminals, but is essential for restricting SLO-1 to presynaptic terminals. In the absence of UNC-2, discrete SLO-1 puncta are often found along the presynaptic regions outside of the presynaptic terminals. Our study highlights the functional coupling of calcium channels and SLO-1 in presynaptic terminals.

### CTN-1/α-catulin organizes SLO-1/BK to form a cluster through interactions with the entire C-terminal region of SLO-1/BK

The BK channel possesses a large intracellular C-terminal region that binds calcium ions. Based on homology to other ion channels and transporters, this region is divided into RCK1 and RCK2 domains. Analogous to the RCK domains found in prokaryotic ion channels and transporters, the RCK1 and RCK2 domains form a closed ring-structure whose diameter changes upon calcium binding, thereby allosterically regulating the transmembrane domains that constitute the channel pore [33]. For this reason, RCK1 and RCK2 domains are called a gating ring. X-ray crystallographic studies of the intracellular C-terminal region of the BK channel showed that even in the absence of the transmembrane domains, RCK1 and RCK2 form the tetrameric top and bottom layers of the gating ring, respectively. Our data have now shown that the interaction between SLO-1 and CTN-1 requires both the RCK1 and RCK2 domains, raising the possibility that CTN-1 interacts with a patch on the outer surface of the RCK1 and RCK2 domains, as opposed to a stretch of amino acids within the two domains. It is also possible that CTN-1 may interact with the RCK1 and RCK2 domains from two neighboring SLO-1 molecules to form a macromolecular complex. How does CTN-1 contribute to SLO-1 function? A previous study using an inside-out patch recording of SLO-1 showed that CTN-1 has no significant effect on SLO-1 channel properties in the presence of different calcium concentrations [13]. Hence, CTN-1 does not appear to alter SLO-1 channel properties. Yet, the loss of *ctn-1* function suppresses the sluggish movement of *slo-1(gf)* and mimics *slo-1* mutant phenotypes, including hyperactive foraging and jerky movement [12]. In addition, our previous electrophysiological recording at the NMJs showed that just as in *slo-1* loss-of-function mutants, evoked amplitude is higher in *ctn-1* mutants than in wild-type animals [12]. We propose that CTN-1 is a calcium nanodomain protein that organizes SLO-1 in the vicinity of VGCCs to form a macromolecular complex that is larger than single tetrameric SLO-1 channels. This organization is likely critical for exposing SLO-1 to the high local calcium concentrations near VGCCs and thus ensures SLO-1 activation when large amounts of calcium ions enter through VGCCs. Consistent with this idea, our analyses in the DA and DB motor neurons revealed that the absence of CTN-1 abolishes the formation of SLO-1 puncta (Figure 2B,C).

### The absence of UNC-2/CaV2 may cause the formation of additional ectopic SLO-1/BK puncta outside of the synaptic terminals through cytoskeletal re-organization

Calcium influx into the active zones of presynaptic terminals via VGCCs triggers the fusion of synaptic vesicles with the plasma membrane. In most neurons CaV2 channels are typically the main VGCCs responsible for this synaptic calcium influx. The absence of CaV2 impairs synchronous, coordinated synaptic vesicle fusion and neurotransmitter release. How can we explain ectopically localized additional SLO-1 puncta in *unc-2* mutants? It is likely that this aberrant localization of SLO-1 results from an altered neurotransmitter release mechanism or compensatory mechanism in *unc-2* mutants. Given that neurotransmitter release is essential for maintaining neural connections and coordinated muscle contractions, the absence of *unc-2* function may lead to two distinct outcomes. One possibility is that the absence of UNC-2 may render synaptic calcium increases dependent on global calcium changes, thereby retaining asynchronous or spontaneous neurotransmitter release to some degree. In this case, SLO-1 is initially localized to the presynaptic terminals, but without proper coupling with calcium channels, some SLO-1 clusters may drift out of the synaptic terminals and disperse throughout the presynaptic regions.

Alternatively, the absence of UNC-2 may cause the recruitment of other calcium channels to, or close to, the presynaptic terminals. In mammals, P/Q-type CaV2.1 is solely responsible for neurotransmitter release at presynaptic motoneuron terminals. In P/Q-type CaV2.1-knockout mice, N-type CaV2.2 and R-type CaV2.3 calcium channels are recruited to the axon terminal regions and replace P/Q-type CaV2.1 function, but these two channels exhibit different localization patterns [34]. R-type CaV2.3 localization closely resembles that of P/Q-type CaV2.1, whereas N-type CaV2.2 localizes away from neurotransmitter release sites and yet contributes to synaptic transmission. Likewise, *C. elegans* appears to have an analogous compensatory mechanism in the absence of UNC-2, the sole CaV2 channel. *unc-2* null mutants, which would be expected to lack synaptic transmission, are severely uncoordinated, but are not completely paralyzed. Furthermore, evoked synaptic currents from the neuromuscular junctions of *unc-2* null mutants are reduced by approximately 60%, but are not eliminated [35]. Hence, it appears that the lack of UNC-2 would cause the recruitment of other calcium channels to, and/or close to, the presynaptic terminals. As in mammals, however, some of the newly recruited calcium channels may not localize exactly to active zones, where UNC-2 normally localizes [36]. Such deviated calcium channel localization in the absence of CaV2 may lead to a partial re-organization of calcium-responsive cytoskeletal proteins that interact with CTN-1. One such potential cytoskeletal protein is F-actin. The structure of F-actin is dynamically regulated by calcium ions, and pharmacological alteration of F-actin structure at presynaptic terminals promotes the efficiency of neurotransmitter release [37,38]. Although F-actin is not known to bind CTN-1, it binds α-catenin and vinculin, two α-catulin homologues [39]. The localization of CTN-1 to perisynaptic sites in addition to the presynaptic terminals is likely to provide additional SLO-1 docking sites.

### Hierarchical organization of SLO-1/BK, CTN-1/α-catulin and DYB-1/dystrobrevin

One intriguing feature of presynaptic organization is that many presynaptic components interact simultaneously with several other components [40]. For instance, the RIM (Rab3-interacting molecules)/UNC-10 protein interacts with MUNC-13, RIM-BP, RAB-3, SYD-2/liprin-α and calcium channels. Intriguingly, SLO-1 is also localized to presynaptic terminals, but its localization is controlled by the hierarchical organization of CTN-1 and DYB-1. SLO-1 is organized as a macromolecular complex and localized by CTN-1, whose localization depends in part on DYB-1. Our data show that whereas the intensity of SLO-1::GFP puncta in *dyb-1* mutants is similar to that of wild-type animals, the density of the puncta is decreased. Thus, we postulate that unlike CTN-1 DYB-1 is not necessary for the formation of macromolecular SLO-1 puncta, rather it stabilizes or maintains the SLO-1 and CTN-1 complex at the presynaptic terminals. Intriguingly, however, DYB-1 is not sufficient for tethering the SLO-1/CTN-1 complex to presynaptic terminals. Supporting this idea, we found that whereas the punctal densities of SLO-1 and CTN-1 along the presynaptic region are increased in *unc-2* mutants, that of DYB-1 remains same in *unc-2* and wild-type animals (Figure 7B). Furthermore, the presynaptic localization of CTN-1 is not completely abolished in *dyb-1* mutants (Figure 3A), suggesting that an additional unknown protein may also interact with CTN-1 and contribute to CTN-1 localization.

Currently, we do not know how DYB-1 localization is controlled. Although STN-1/syntrophin is a potential interacting protein for DYB-1, *stn-1* null mutants do not exhibit significantly altered SLO-1 localization. Thus, we postulate that DYB-1 synaptic localization is redundantly controlled by STN-1 and other unidentified proteins, or that it may not involve STN-1 at all.

### Calcium channel localization and SLO-1/BK localization may be regulated independently

Previous studies using co-immunoprecipitation and physiological analyses suggested that VGCCs and BK channels are co-assembled in calcium nanodomains [20,41]. These results imply that the localization of BK channels and VGCCs is dependent on each other’s presence. However, our study shows that the absence of UNC-2 does not impede SLO-1 localization to presynaptic terminals, although the strict localization of SLO-1 to presynaptic terminals is compromised. Furthermore, the presynaptic localization of UNC-2 was not altered in *slo-1* null mutants. In addition, the absence of SLO-1 puncta at presynaptic terminals in *ctn-1* mutants does not endow these animals with *unc-2*-like uncoordinated movement [12]. Together, our study indicates that the localization of UNC-2 and SLO-1 is established independently.

## Conclusion

The SLO-1 potassium channels distinctively form organized clusters at presynaptic terminals. Two cytoskeletal proteins, CTN-1 and DYB-1, are crucial for normal SLO-1 cluster formation; CTN-1 directly organizes SLO-1 clusters, and DYB-1 stabilizes or maintains the complex of CTN-1 and SLO-1 complex through the interaction with CTN-1. UNC-2 calcium channels that provide calcium ions for neurotransmitter release influence the localization specificity of the SLO-1 channel clusters. However, SLO-1 and UNC-2 channel clusters are formed independently from each other. Thus, SLO-1 channels are closely placed near UNC-2 calcium channels through an indirect mechanism where CTN-1 plays a role.

## Methods

### C. elegans maintenance and strains

*C. elegans* strains were cultured and maintained on nematode growth medium (NGM)-agar plates using standard methods at 20°C. The following strains were used in this study: wild-type N2, unc-13(e51), unc-2(e55), unc-2(zf35gf), slo-1(eg142), slo-1(ky399gf), dyb-1(cx36), ctn-1(eg1167), stn-1(tm795) and dgk-1(nu62).

### Microinjection and transformation

Microinjection was performed according to Mello et al. [42]. We injected approximately 0.1 nl of DNA mixtures (a GFP- or mCherry-tagged gene under the indicated promoter, a marker DNA and pBluscriptSK) at the final concentration of 100 ngμl^−1^ into the distal region of the gonad syncytium. From progeny of the injected animal, we selected transgenic lines that stably transmit extrachromosomal arrays to subsequent generations.

### Extrachromosomal or integrated chromosomal arrays

When the comparison of the punctal intensity and density between two different genotypes is necessary, we integrated extrachromosomal arrays from the chosen transgenic lines into the genome with a method using ultraviolet/trimethylpsoralen [43]. The following list is the extrachromosomal or chromosomally integrated arrays used in our study.

*cimIs10[punc-129slo-1a::GFP]*: The plasmid backbone was derived from pPD118.20. The 2.4 kb HindIII-NotI fragment (*myo-3* promoter) of pPD118.20 was replaced with 2.4 kb-PCR-amplified *unc-129* promoter sequence that drives expression in a subset of DA and DB neurons. The N-terminal *slo-1a* cDNA (channel and RCK1 domain) was subcloned into the NotI and KpnI sites upstream of the GFP sequence of *Punc-129GFP*, and the remaining C-terminal *slo-1a* cDNA (RCK2 domain) was subcloned in frame into the NheI site downstream of the GFP sequence. The resulting construct was injected at 10 ngμl^−1^ along with the marker *ttx-3p::mCherry* (30 ngμl^−1^).

*cimIs8 [punc-129GFP::ctn-1]*:*ctn-1* cDNA (2.3 kb) was subcloned in frame into the EcoRI site of *punc-129GFP*. The resulting construct was injected at 1 ngμl^−1^ along with the marker *ttx-3p*::mCherry (30 ngμl^−1^).

*cimIs15[punc-129GFP::dyb-1]*: *dyb-1* cDNA (1.7 kb) was subcloned in frame into the NheI site of *punc-129GFP*. The resulting construct was injected at 2 ngμl^−1^ along with the marker *ttx-3p*::mCherry (30 ngμl^−1^).

*cimIs14[punc-129mCherry::rab-3]*: The GFP sequence from *punc-129*GFP was replaced with the mCherry(Opt) sequence and *rab-3* cDNA (Open Biosystems) was subcloned in frame to the NheI site using the Gateway cloning system (Life technologies). The resulting construct was injected at 3 ngμl^−1^ along with the marker *odr-1p*::GFP (30 ngμl^−1^).

*cimIs25[punc-129GFP::unc-2]*: The *odr-3* promoter sequence (FseI-AscI fragment) from the *odr-3p*GFP::*unc-2* construct (a kind gift from Cori Bargmann) was replaced with the *unc-129* promoter. The resulting construct was injected at 20 ngμl^−1^ along with the marker *ttx-3p*::mCherry (30 ngμl^−1^).

*cimEx51[punc-129elks-1::mCherry]:* The genomic *elks-1* DNA was amplified by PCR and subcloned to the NotI site present between mCherry(Opt) coding sequence and the *unc-129* promoter in a vector derived from pPD118.20. The resulting construct was injected at 0.5 ngμl^−1^ along with the marker *odr-1p*::mCherry (30 ngμl^−1^).

*cimEx52*[*punc-129slo-1a::GFP, punc-129mCherry::CTN-1]: ctn-1* cDNA (2.3 kb) was subcloned in frame into the EcoRI site of *punc-129mCherry*. The resulting construct was injected at 1 ngμl^−1^ together with *punc-129slo-1a::GFP* (10 ngμl^−1^) and the marker *ttx-3p*::mCherry (30 ngμl^−1^).

### Yeast two-hybrid analysis

Yeast two-hybrid assays were performed using the Matchmaker GAL4-based yeast two-hybrid system (Clontech). Various DNA fragments derived from *ctn-1* were subcloned into the pGBKT7 vector in frame with the GAL4 DNA-binding domain, and the resulting constructs were transformed into the Y187 yeast strain. Various *slo-1* cDNA fragments were subcloned into the PGADT7 vector in frame with the GAL4 activation domain, and the resulting constructs were transformed into the Y2H Gold strain. Mating was performed by inoculating and growing appropriate yeast colonies in YPD medium, followed by the selection of diploids that showed positive two-hybrid interactions on quadruple dropout plates (−histidine, −alanine, −leucine, and -tryptophan) containing 40 μg/ml X-α-Gal. Empty vectors served as negative controls, and constructs were tested for autoactivation.

### Measurement of the locomotory speed

To measure the speed of the animals, twelve to fifteen age-matched hermaphrodites (30 hr after L4 stage) for a given genotype were first placed on an unseeded NGM plate for 15 min. This process effectively removed bacteria attached to the animals. The animals were then transferred to the inside of a copper ring embedded in a NGM plate. To avoid any potential variations due to the differences in NGM plates, we simultaneously measured the speeds of three different genotypes in a single plate embedded with three copper rings. Video frames of three different genotypes were acquired with a dissecting microscope equipped with Go-3 digital camera (QImaging) for 2 min with a 500 ms interval and 20 ms exposure. We measured the average speed of the animals using Track Objects in ImagePro Plus (Media Cybernetics).

### Microscopic imaging

Animals in the first day of adulthood (18 hr after L4 crescent stage) were immobilized on a thin 2% agarose pad with a 6 mM levamisole (Sigma-Aldrich) solution in M9 buffer. After complete immobilization (approximately 10 min), a coverslip was placed on top of the agarose pad and the four corners were sealed with nail polish. Animals were imaged within 40 min of mounting. Images were acquired using a 63×/1.4 numerical aperture (N.A.) or 100×/1.4 N.A. objective on a Zeiss inverted microscope (Axio Observer Z1) equipped with an HXP120 metal halide illuminator that produces a more stable output than conventional mercury-based illuminators. We captured 12-bit images of Z sections (0.2 μm × 20 slices) with a CoolSNAP HQ2 interline CCD camera (Photometrics) controlled by Metamorph (Molecular Devices Inc.) software. In many cases, maximum intensity projections of image stacks were produced to quantify the fluorescence images. We included wild-type controls during each image acquisition session to ensure that any differences observed in the mutants were not due to changes in illumination. Image acquisition conditions such as fluorescence intensity, exposure time and gain were set identically for a given integrated fluorescent marker.

### Image processing and quantification

We typically observed more than 100 animals for each genotype and obtained similar results within a given genotype. For intensity quantification purpose, however, we used images acquired from animals whose dorsal cord region, an above region between DB6 and DA5 cell bodies, directly faced the objective. Due to the various background levels in the images, the imaging software threshold feature was not effective in discerning fluorescence puncta from the background. Hence, we wrote a custom MATLAB-based program, dotGUI, that allows thresholding only a region of interest. Images were rotated to position the dorsal axon terminal containing fluorescent puncta horizontally. We then selected a region of interest by drawing a rectangular box that surrounds the fluorescent puncta. We used a threshold operation for pixel intensities greater than the 50^th^ percentile of the intensity distribution taken from the user-generated region of interest. Punctal distance was derived from the pixel distance between the centroids of the two nearest neighbor puncta. Intensity values (maximum and mean intensity) from puncta were obtained by subtracting the 10-percentile intensity value of the background levels in the region of interest. This custom software has a feature that allows a user to add or remove certain dots that are below the median intensity values. Because the size of newly added dots was arbitrarily set to 9 pixels, we excluded punctal size from our analysis.

To measure the average presynaptic fluorescence intensity, we used a line-scanning method in Metamorph software. Specifically, we first measured the average pixel intensity of 150 pixel length from the presynaptic region that includes presynaptic terminals, and then subtracted the adjacent average background pixel intensity. The resulting adjusted average pixel intensity is called the average presynaptic fluorescence intensity.

## Additional files

Additional file 1: Figure S1. SLO-1 and CTN-1 co-localize in the presynaptic region of DA and DB motor neurons. Transgenic animals expressing GFP-tagged SLO-1 and mCherry-tagged CTN-1 under the control of the *unc-129* promoter that drives expression in DA and DB motor neurons were used for assessing co-localization of SLO-1 and CTN-1. Scale bar, 5 μm. Additional file 2: Figure S2. The overall SLO-1 fluorescence intensity along the presynaptic region is not different in wild-type, *ctn-1* and *dyb-1* mutant animals. The data is presented as mean ± SEM and analyzed by One-way ANOVA with Dunnett’s multiple comparisons (*ctn-1* and *dyb-1* are not significantly different from wild-type). Additional file 3: MP4. A movie of *unc-2(gf), slo-1(gf)* and *slo-1(gf);unc-2(gf)* double mutants. The video is accelerated 10 times. Additional file 4: Figure S4. The expression of SLO-1::GFP remain same in *unc-2* mutants. Decreased punctal distance in *unc-2* mutants is not due to an altered SLO-1::GFP levels in DA and DB neurons, as the level of SLO-1::GFP expression is indistinguishable in wild-type and *unc-2* animals. The average intensities of individual nuclei are not different in wild-type (977.5 ± 44.55) and *unc-2* (949.7 ± 52.58) animals (Student t-test, n = 10, *p* = 0.69). The data (artificial intensity units) represent mean ± SEM. The scale bar represents 5 μm. Additional file 5: Figure S5.
*unc-2* mutants exhibit a slight reduction in SLO-1 punctal intensity without change in overall SLO-1 levels in the presynaptic region. Average maximum punctal intensity (mean ± SEM) was calculated by subtracting local average intensity values from the peak values (n = 10, * *p* = 0.0319, t-test). Average presynaptic fluorescence intensity (mean ± SEM) was calculated by subtracting the adjacent average background pixel intensity from the average pixel intensity of 150 pixel length from the presynaptic region, which includes presynaptic terminals and inter-punctal regions (n = 10, n.s. *p* = 0.1114, t-test).
