# Supplementary figures and images for: Presynaptic BK channel localization is dependent on the hierarchical organization of alpha-catulin and dystrobrevin and fine-tuned by CaV2 calcium channels
Source: BMC Neurosci. 2015 Apr 24;16:26. doi: 10.1186/s12868-015-0166-2 (PMC4411755; doi:10.1186/s12868-015-0166-2)

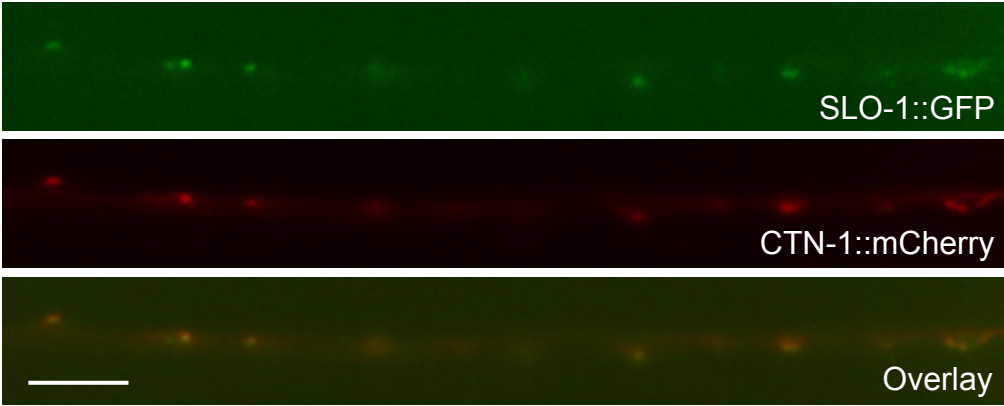

Supplement: Additional file 1: Figure S1. — SLO-1 and CTN-1 co-localize in the presynaptic region of DA and DB motor neurons. Transgenic animals expressing GFP-tagged SLO-1 and mCherry-tagged CTN-1 under the control of the unc-129 promoter that drives expression in DA and DB motor neurons were used for assessing co-localization of SLO-1 and CTN-1. Scale bar, 5 μm. [file 12868_2015_166_MOESM1_ESM.pdf]

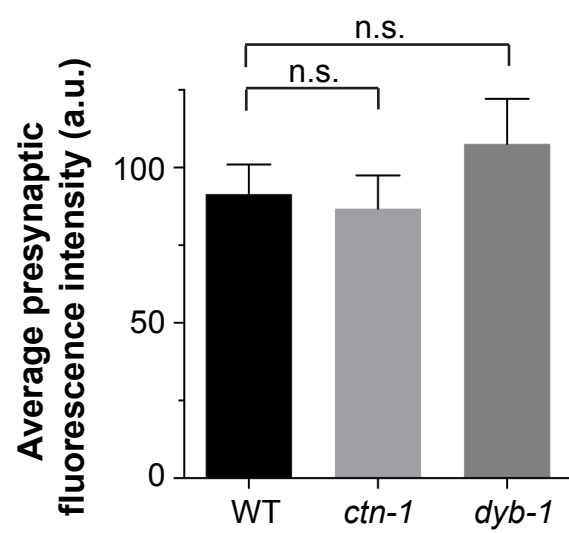

Supplement: Additional file 2: Figure S2. — The overall SLO-1 fluorescence intensity along the presynaptic region is not different in wild-type, ctn-1 and dyb-1 mutant animals. The data is presented as mean ± SEM and analyzed by One-way ANOVA with Dunnett’s multiple comparisons (ctn-1 and dyb-1 are not significantly different from wild-type). [file 12868_2015_166_MOESM2_ESM.pdf]

**A**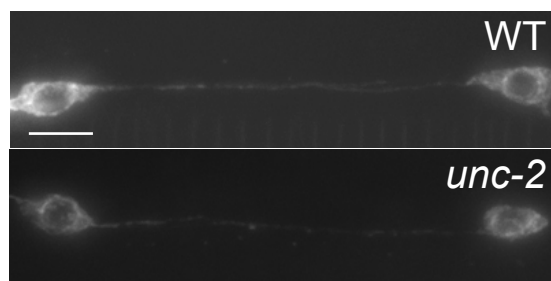**B**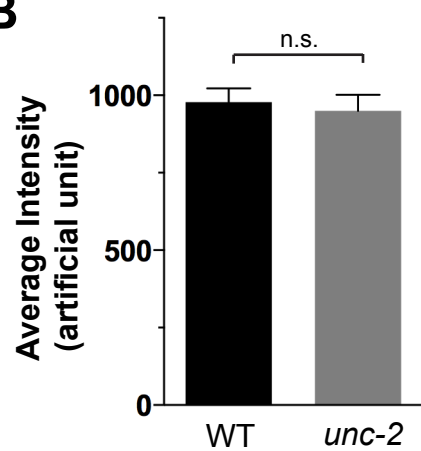

Supplement: Additional file 4: Figure S4. — The expression of SLO-1::GFP remain same in unc-2 mutants. Decreased punctal distance in unc-2 mutants is not due to an altered SLO-1::GFP levels in DA and DB neurons, as the level of SLO-1::GFP expression is indistinguishable in wild-type and unc-2 animals. The average intensities of individual nuclei are not different in wild-type (977.5 ± 44.55) and unc-2 (949.7 ± 52.58) animals (Student t-test, n = 10, p = 0.69). The data (artificial intensity units) represent mean ± SEM. The scale bar represents 5 μm. [file 12868_2015_166_MOESM4_ESM.pdf]

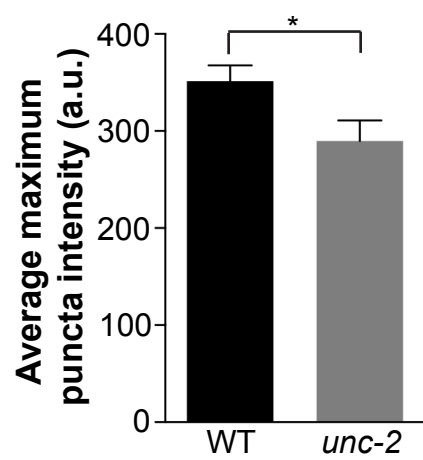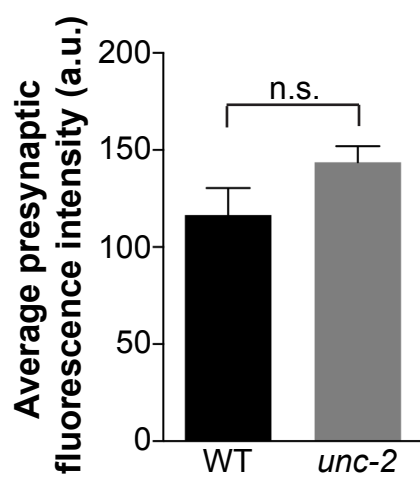

Supplement: Additional file 5: Figure S5. — unc-2 mutants exhibit a slight reduction in SLO-1 punctal intensity without change in overall SLO-1 levels in the presynaptic region. Average maximum punctal intensity (mean ± SEM) was calculated by subtracting local average intensity values from the peak values (n = 10, * p = 0.0319, t-test). Average presynaptic fluorescence intensity (mean ± SEM) was calculated by subtracting the adjacent average background pixel intensity from the average pixel intensity of 150 pixel length from the presynaptic region, which includes presynaptic terminals and inter-punctal regions (n = 10, n.s. p = 0.1114, t-test). [file 12868_2015_166_MOESM5_ESM.pdf]
